# Nanomaterial‐Based Inkjet Printing for Electrochemical Sensing

**DOI:** 10.1002/smll.202513028

**Published:** 2026-01-08

**Authors:** David Panáček, Massimo Urban, Alessandro Silvestri, Ivan Dědek, Martin‐Alex Nalepa, Arben Merkoçi, Maurizio Prato, Michal Otyepka

**Affiliations:** ^1^ Czech Advanced Technology and Research Institute (CATRIN) Palacký University Olomouc Olomouc Czech Republic; ^2^ Nanotechnology Centre Centre for Energy and Environmental Technologies VSB–Technical University of Ostrava Ostrava Czech Republic; ^3^ Catalan Institute of Nanoscience & Nanotechnology ‐ ICN2 (BIST and CSIC), Campus UAB, 08193 Bellaterra (Barcelona) Spain; ^4^ Department of Molecular Sciences and Nanosystems Ca’ Foscari University of Venice Venezia Italy; ^5^ Department of Physical and Macromolecular Chemistry Faculty of Science Charles University Prague Czech Republic; ^6^ Catalan Institution for Research and Advanced Studies (ICREA) Passeig de Lluís Companys 23 Barcelona Spain; ^7^ Center for Cooperative Research in Biomaterials (CIC BiomaGUNE) Basque Research and Technology Alliance (BRTA) Donostia‐San Sebastián Spain; ^8^ Ikerbasque Basque Foundation for Science Bilbao Spain; ^9^ Department of Chemical and Pharmaceutical Sciences University of Trieste Trieste Italy; ^10^ IT4Innovations VSB–Technical University of Ostrava Ostrava Czech Republic

**Keywords:** functionalization, inkjet, monitoring, nanomaterials, sensor

## Abstract

Inkjet printing (IJP) has emerged as a transformative technology for printed and flexible electronics, redefining electrode engineering for (bio)chemical sensing. It enables maskless, picoliter‐scale, additive deposition with high spatial precision, uniformity, and material efficiency. We provide a comprehensive overview of IJP as both a fabrication and post‐fabrication functionalization platform for electrochemical working electrodes and fully printed devices. We integrate advances in ink formulation, jetting behavior, and substrate interactions with performance metrics such as layer thickness, roughness, electrochemical surface area, sensitivity, detection limit, and reproducibility. Comparative analyses with drop‐casting and screen‐printing highlight IJP's advantages in reproducibility, scalability, and material economy. Particular emphasis is placed on nanomaterial‐ and bioink‐based systems, including carbon nanomaterials, MXenes, and hybrid inks, where controlled deposition governs electrode functionality. We also discuss emerging opportunities in hybrid architectures, reactive printing, and sustainable approaches using biodegradable substrates and water‐based inks. Finally, we outline a roadmap toward automated, digitally controlled, and environmentally responsible manufacturing of customizable sensors for wearable, biomedical, food, and environmental applications. Collectively, these developments position inkjet printing as an enabling framework for the next generation of intelligent, reproducible, and sustainable sensing technologies.

## Graphical abstract

Inkjet printing provides a digitally controlled, versatile, and highly reproducible method for fabricating and functionalizing sensing devices. This Perspective highlights how homogeneous, material‐efficient deposition enables precise patterning of functional inks and scalable integration of sensors for chemical and biological detection. The discussion outlines opportunities and challenges of IJP in next‐generation sensing technologies.

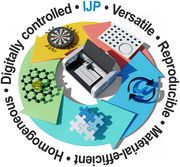

## 1 Introduction

Printing technology, in the context of electronic and sensor development, refers to the additive patterning of functional materials onto a wide variety of substrates using digitally or physically guided deposition methods [1, 2, 3, 4]. Unlike traditional subtractive techniques, such as photolithography, which require multistep processes involving masking, etching, and cleaning after UV or electron beam exposure [5] in a controlled environment of cleanrooms, printing offers a more cost‐effective and environmentally friendly alternative [6, 7]. Inkjet printing (IJP), recognized as one of the most cost‐effective techniques for device prototyping and scalable fabrication [8], emerges as a precise and versatile approach, offering non‐contact, digital, and additive deposition of materials with micrometer‐scale resolution [9, 10]. Initially developed for graphic applications, IJP technology has become a fundamental technique in materials science and, similar to screen printing (SP), has attracted significant attention from the scientific community, covering applications ranging from sensors [11, 12, 13], including strain [14], gas/pressure sensors [15], and nature‐inspired sensing systems [16], to energy [17, 18] and biotechnology [19, 20, 21]. IJP, compared to conventional SP, eliminates the need for masks or meshes, enabling direct transfer of patterns from digital designs to substrates [22, 23]. IJP enables highly efficient material utilization, reducing consumption to the microgram scale [24] through precise deposition and negligible losses, which in turn markedly decreases production costs. Nevertheless, SP remains the technique of choice for large‐scale industrial production, although it is generally less flexible from a design‐to‐device perspective.

**FIGURE 1:**
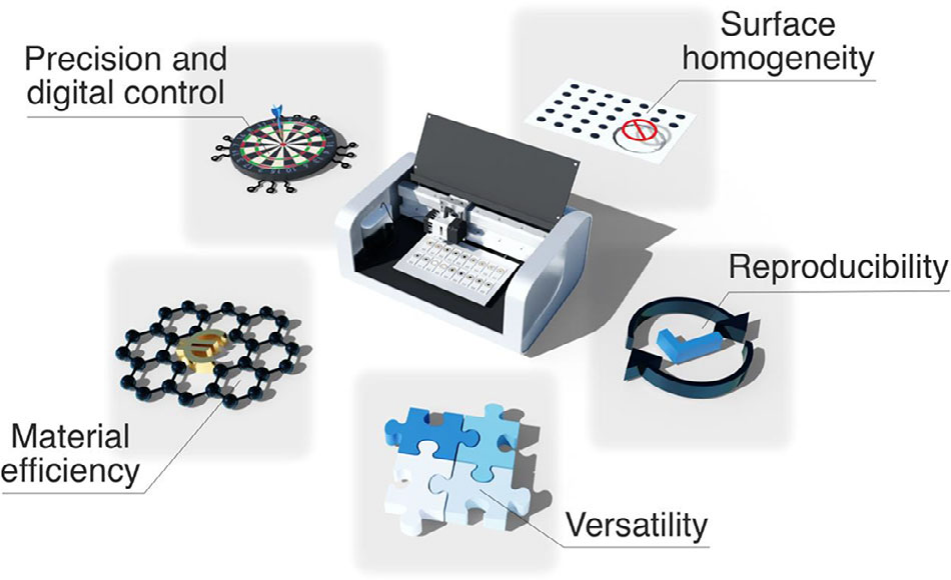
Benefits of IJP for sensor fabrication and functionalization.

In the electrochemical sensor development, the core functional element is the working electrode (WE), where surface chemistry, morphology, and electronic properties determine the sensitivity, selectivity, and stability of the sensing system [25, 26]. Although bare electrodes serve as the structural substrate and electrical base [27, 28], the selective recognition function originates from the functional layer [29, 30, 31]. Traditionally, the functionalization layer is produced using physical or chemical deposition techniques, including drop‐casting (DP) [32], electro‐polymerization (EP) [33], or spin‐coating [34]. Despite their widespread use, these methods are inherently constrained by non‐uniform material distribution and limited reproducibility (in the case of DP), or poor scalability for high‐throughput manufacturing [35] (e.g., in EP and spin coating). DP, widely employed in both academic research and commercial applications for fabricating customized selective sensors, suffers from significant spatial inhomogeneity due to the coffee‐ring effect, rapid solvent evaporation, and unregulated droplet spreading. These drawbacks result in inconsistent coverage of the electroactive surface, leading to poor signal reproducibility [36]. EP offers better control of material distribution across the entire electrode area but often requires fine‐tuning and specialized equipment (e.g., precise potentiostatic or galvanostatic control, synchronized multi‐electrode configurations, and tightly regulated temperature) [37]. Moreover, EP is sensitive to electrolyte composition and presents issues in miniaturized, multiplexed, or flexible sensor systems [38]. In response to these limitations, IJP has emerged as a highly promising strategy for the post‐fabrication functionalization of electrodes [24], and it could play a key role, particularly when expensive nanomaterials or sensitive biomolecules need to be deposited on the WE in a uniform and precise manner. Unlike roll‐to‐roll compatible methods such as SP, which optimize device macroarchitecture [39, 40], IJP enables picoliter‐scale, high‐precision deposition of functional materials when required [9]. This capability is particularly advantageous when developing small devices [41] or functionalizing small surfaces such as WEs, where coating accuracy is critical [42]. It also offers additional benefits, including reproducibility, material efficiency, and surface homogeneity (Figure 1).

This review highlights the potential of IJP as an efficient platform for precision surface engineering of electrodes, redefining how sensors can be functionalized in various fields. In addition, it provides an in‐depth analysis of the current state‐of‐the‐art, identifies the parameters that govern the performance of functionalized layers, and presents future directions for this method, such as the integration of IJP directly into automated, high‐throughput, digitally controlled sensor assembly lines.

## 2 Inkjet Printing for Sensor Fabrication

For over two decades, printed electronics has been investigated as a sustainable alternative to conventional photolithography‐based fabrication [43, 44]. Printing methods enable device patterning on flexible, silicon‐free substrates, creating new opportunities for applications that can surpass those achievable with the traditional methods. These methods can be broadly divided into contact and non‐contact methods, based on whether the substrate comes into direct contact with the ink‐dispensing unit [45]. Contact methods include SP, nanoimprinting, flexographic printing, and gravure printing, where ink is transferred to the substrate using a mask, mold, or a stamp. In contrast, non‐contact methods such as IJP, extrusion‐based (3D) printing, and aerosol jet printing deposit ink without physical contact, relying on the precise control of droplet ejection.

Among these methods, SP and IJP have been the most widely adopted for electrochemical sensor fabrication over the past decades. Their success is driven by low cost, versatility, relatively simple equipment, and the availability of a variety of functional inks [46, 47]. SP remains the preferred technique in many academic and industrial settings, but interest in IJP has also increased. IJP is now viewed as a highly adaptable approach, offering digital, maskless, and additive fabrication with minimal material waste and broad compatibility with conductive and functional nanomaterials [23, 48]. The significance of IJP extends well beyond its origins in graphic printing. Its capability to digitally deposit colloidal solutions of conductive materials soon established it as a transformative technique for printed electronics. Colloidal dispersions of nanomaterials can be synthesized via different routes, with tunable concentrations and rheological properties tailored to meet IJP requirements [3, 49].

In sensor research, IJP has enabled the direct fabrication of electrodes from a wide range of inks. Silver nanoparticles are frequently used for reliable electrical contacts and interconnects [50]. Gold nanoparticles [51], carbon nanotubes (CNTs) [52], conductive polymers [53], and graphene derivatives [24] provide electroactive interfaces that define the functional sensing surface. A key advantage of IJP is its ability to combine multiple ink formulations within a single device, thereby reducing fabrication complexity and enabling multifunctional architectures, such as sensor arrays [54]. Although industrial adoption is still limited, interest is steadily increasing. Fully inkjet‐printed electrodes are still uncommon commercially, mainly due to technical bottlenecks that must be addressed for large‐scale production.

IJP offers several key advantages compared to other printing techniques. It is a maskless, digital process where patterns are defined directly from a digital design, enabling rapid customization and real‐time prototyping. Ink waste is minimal because droplets are deposited only where required, minimizing waste and improving material efficiency as a result. Moreover, the additive nature of IJP supports multilayer structures and the integration of diverse materials without additional tooling or substrate changes. This is an important distinction from other printing methods, as modern IJP printheads can handle multiple inks simultaneously, reducing the need for sequential processing and enabling integration of conductive, insulating, and functional layers [54]. For example, even office printers can handle 4–8 colors, with professional systems reaching up to 12 different colors. Together, these advantages make IJP particularly suited for applications requiring both design flexibility and material diversity, positioning it as a key enabling technology for customizable sensing platforms.

### 2.1 Challenges and Gaps in Inkjet Sensor Fabrication

**FIGURE 2:**
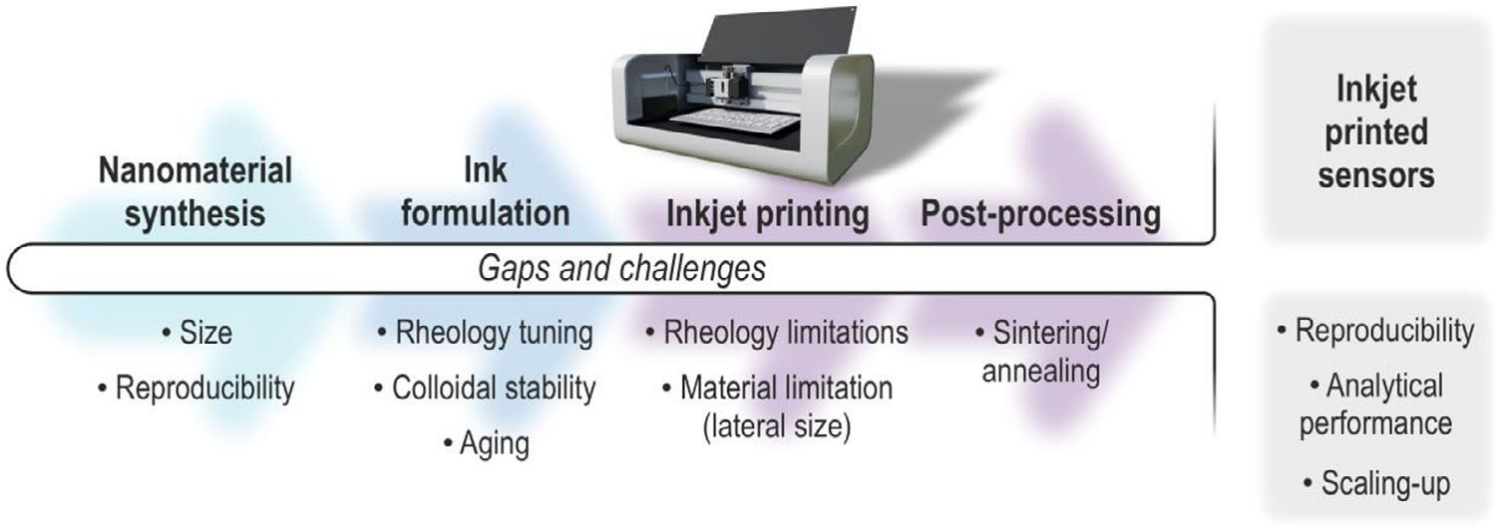
Current challenges in the fabrication of electrochemical sensors using IJP.

Despite these strengths, several barriers remain before IJP can fully transition from laboratory prototyping to industrial‐scale manufacturing and achieve broad adoption in electrochemical sensing (Figure 2). Key challenges lie in ink formulation and reproducibility, long‐term ink stability and cartridge compatibility, and the need for efficient post‐processing methods. In addition, intrinsic material limitations, such as particle size requirements for nanomaterial‐based inks, continue to constrain performance and scalability.

#### 2.1.1 Ink Formulation and Reproducibility

Reliable device performance requires standardized, reproducible workflows spanning nanomaterial synthesis, ink preparation, printing, and post‐treatment. While the discovery of new materials remains important, greater emphasis should be placed on optimizing existing nanomaterials for printability and reproducibility. Variability in nanoparticle size, surface chemistry, and capping agents often translates into inconsistent electrical and electrochemical behavior, which ultimately affects biorecognition and sensing. Achieving consistent ink formulation is therefore essential for the broader adoption of fully inkjet‐printed sensors in analytical applications.

#### 2.1.2 Stable Droplet Generation and Jetting

Another critical challenge lies in the dynamics of fluid behavior and droplet generation. Nanomaterial‐based inks must exhibit reliable jetting performance, characterized by stable droplet formation, optimized waveform control, and consistent behavior over time. Although suitable printing parameters, such as driving voltage and waveform shape, can often be identified through short optimization procedures, these parameters tend to drift during prolonged operation. Continuous fine‐tuning by the operator is often required to maintain consistent print quality. This instability arises from the intrinsic variability of nanomaterial inks, batch‐to‐batch differences, and even factors such as printhead aging or inadequate maintenance. While such manual optimization is manageable at a laboratory scale, it poses significant challenges for industrial translation, where reproducibility and automation are essential. As a result, drop stability and long‐term jetting reliability remain key bottlenecks limiting the widespread adoption of inkjet printing for sensor fabrication.

#### 2.1.3 Ink Stability and Printhead Compatibility

In research, inks are often freshly prepared and loaded directly into cartridges. In industrial scenarios, however, inks must remain stable inside printheads for extended periods without clogging or degradation. Colloidal stability, interactions with dispersants or stabilizers, and long‐term cartridge compatibility remain underexplored. Addressing these issues is critical for reliable high‐throughput production. A detailed understanding of how dispersion stability evolves during storage and within the printing system will be essential for scale‐up. Studies should assess not only material degradation but also interactions between inks and the printer hardware to identify critical steps in the fabrication pipeline.

#### 2.1.4 Ink‐Substrate Compatibility

A critical and often overlooked aspect in sensor fabrication with IJP is the appropriate selection of the substrate and its interaction with the ink. Substrates can vary significantly in their physical and chemical properties, such as chemical inertness, hydrophobicity, rigidity, or porosity, even within a specific material class (e.g., plastics or papers) [55, 56].

All these properties ultimately affect the entire fabrication process, in some cases, requiring additional steps, such as preparing the surface to make it more hydrophilic or rough [57, 58]. This process, known as priming in the graphic industry, is a crucial step in enhancing ink adhesion and substrate wetting, resulting in more vibrant colors for graphic patterns or, in the case of nanomaterials, stronger adhesion and more uniform surface coverage. Other strategies, such as controlling the ink's drying speed by heating the substrate or working in humidity‐controlled environments, have also proven effective in improving film quality, although they can add complexity during the optimization phase. More emphasis should be placed on studying the materials used as substrates, understanding the fundamental interactions with the inks, and the implications it has on the manufacturing process of the printed sensors.

#### 2.1.5 Post‐Processing

The deposition of functional materials is only one step in producing a complete sensor. Post‐printing treatments, such as sintering, curing, or deposition of dielectric layers, are essential but can create obstacles due to temperature or chemical incompatibilities between the inks and substrates. Low‐temperature and rapid post‐processing methods (e.g., photonic curing, plasma treatments, chemical sintering) are particularly important for enabling printing on temperature‐sensitive and lightweight substrates.

Depending on the specific architecture and the intended use of the printed sensor, additional stabilization layers may be required, for instance, in the presence of biolayers, to protect sensor functionality and ensure long‐term storage and stability.

#### 2.1.6 Material Limitations

Two‐dimensional materials, such as graphene derivatives or MXenes, offer exceptional promise for sensing [59], but their use is constrained by particle size requirements. A common guideline is that the particle size must be smaller than 1/50 or 1/25 of the nozzle diameter, practically corresponding to mean dimensions below 1 µm [60], which limits the use of larger flakes that often exhibit superior electrical performance. Emerging strategies to broaden the printable size range of nanomaterials could unlock new opportunities for high‐performance sensors, as shown in recent studies challenging these common guidelines [60].

### 2.2 Economic Considerations in Scaling Inkjet Printing for Sensor Fabrication

From an industrial manufacturing perspective, comparing the costs of IJP and SP requires evaluating not only equipment price but also workflow characteristics and long‐term operational expenses. Among commonly used fabrication methods, SP and IJP occupy a similar space: both rely on functional inks, both enable pattern deposition on flexible substrates, and both are significantly more affordable and operationally simpler than photolithography. For this reason, they represent the most relevant economic competitors in the context of printed electrochemical sensors.

Both techniques can employ different types of nanomaterial‐based inks, as previously discussed; yet, the rheological requirements differ substantially. SP requires highly viscous pastes, whereas IJP necessitates tightly controlled low‐viscosity, low‐surface‐tension formulations. Despite this constraint, IJP offers a major economic advantage in virtually zero ink waste. Because droplets are deposited only where needed, expensive functional inks, particularly noble‐metal nanoparticle formulations, are used far more efficiently, lowering material consumption and, in some cases, making it economically feasible to incorporate premium materials that would be prohibitively costly in SP. For example, several reports demonstrate that fully inkjet‐printed electrodes can be produced at lab‐scale costs of less than one US dollar per device [24, 61, 62]. The digital nature of IJP eliminates the need to fabricate and replace printing masks, allowing for rapid design iterations without additional material or operational costs. This feature substantially reduces the initial investment and makes IJP an attractive option for laboratories, startups, and low‐volume production environments. Furthermore, the ability to change sensor architecture or materials in real time, using only a digital design file, directly translates into operational flexibility that traditional SP workflows cannot match.

Nevertheless, these benefits of IJP must be weighed against the strengths of industrial SP, which provides superior throughput and reduced unit costs for high‐volume manufacturing. While a single IJP printer can dynamically modify designs, it typically prints more slowly than SP lines and requires more stringent maintenance to prevent nozzle clogging or printhead degradation. These factors ultimately influence the per‐unit cost at industrial scales and must be considered when assessing the overall economic feasibility of IJP‐based sensor production. Nevertheless, with the never‐ending progression of machine engineering, the new generation of printing units can efficiently print hundreds of pages per minute, and can even be integrated into roll‐to‐roll workflows, making inkjet printing a real option for the next generation of large‐scale sensor fabrication.

## 3 Inkjet Printing as a Precision Functionalization Tool for Electrochemical Interfaces

### 3.1 Principles of Inkjet Printing in Working Electrode Engineering

IJP, particularly in its drop‐on‐demand (DOD) configuration, operates by ejecting picoliter‐scale ink droplets onto the substrate with micrometer precision [9], enabling non‐contact deposition of digitally defined patterns [63]. WEs typically require modification with redox‐active materials [64], electrocatalysts [65], or biomolecules [66]. Unlike conventional coating methods, IJP enables selective and uniform deposition of these materials directly onto the functional core of the sensor (i.e., WE), without altering other sensor components or the overall device geometry [24]. This becomes particularly relevant when fabricating miniaturized sensors, multiplexed electrode arrays, and devices made on flexible or curved substrates, where excessive deposition, leakage, or mechanical disruption could compromise the overall integrity. Precise control over droplet size, spacing, and number of layers further enables tuning of properties such as film thickness, porosity, and roughness, which directly influence electrochemical performance.

Carbon nanomaterials are particularly attractive for these purposes due to the combination of tunable surface chemistry and favorable charge transport characteristics. While some carbon nanostructures offer abundant functional groups for molecular recognition and immobilization [67], others facilitate efficient electron transfer [68]. Ideally, materials that integrate both features can serve as standalone inks for the fabrication of WEs. Current research efforts, therefore, aim to position carbon‐based nanomaterials as sustainable alternatives to the well‐established metal nanoparticle inks [69]. Among the earliest and most widely studied carbon‐based inks are 1D and 2D nanomaterials such as CNTs [70], graphene [71], and reduced graphene oxide [72]. These materials have primarily been employed to enhance conductivity, and while functionalized carbon inks offer additional chemical versatility, their formulation is complicated by reduced colloidal stability and a tendency to aggregate. Printable graphene‐oxide‐based inks have been used in sensor fabrication [73], but examples of printable graphene inks incorporating functional groups suitable for biofunctionalization remain limited. A recent development in this area is a water‐based ink composed of nitrogen‐doped carboxylated graphene (NGA), which contains approximately 13 wt.% carboxylic acid groups [24]. This high degree of functionalization could potentially enable the covalent attachment of biomolecules via EDC/NHS chemistry, facilitating the single‐step printing of biosensing interfaces.

In addition to graphene derivatives, other 2D materials, such as transition metal dichalcogenides (e.g., MoS_2_, WS_2_) [3] and MXenes [74] have also been formulated into inks for IJP and applied for sensing applications. These materials offer complementary electrochemical properties, including redox activity and ion intercalation capacity, although their integration into robust inks presents similar challenges with respect to stability and nozzle compatibility [75]. A particularly promising direction is the formulation of biofunctional inks, or “bioinks”, that incorporate biorecognition elements directly into the ink [76]. These enable the additive fabrication of sensing layers without requiring post‐printing modification. Examples include inks containing enzymes [77, 78], aptamers [79, 80], or bacteriophages [81]. Currently, such bioinks are primarily used to functionalize prefabricated conductive surfaces rather than serving as independent sensing materials. A key future step toward their broader use in electrochemical sensors will be the development of standalone bioinks—formulations that simultaneously meet printing requirements (e.g., viscosity, surface tension, droplet formation), preserve the activity of biorecognition components, and provide sufficient conductivity for direct electrode fabrication [82]. Achieving this balance demands precise optimization of ink formulations and printing parameters and remains an active area of research.

**FIGURE 3:**
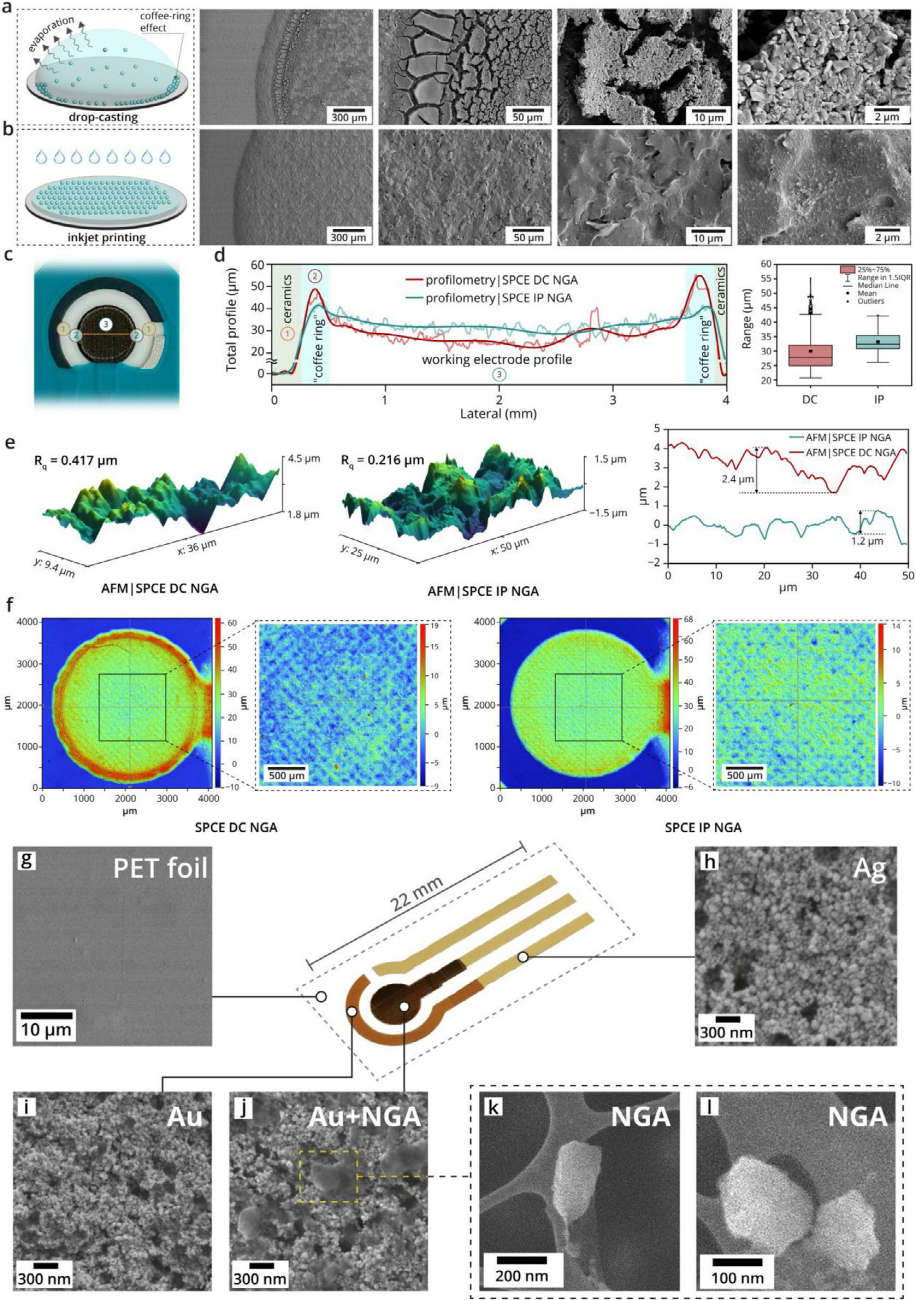
Microscopic analyses illustrating the uniformity of inkjet‐deposited functional layers. (a) Schematic illustration of the DP process and corresponding SEM images of SPCEs modified using this technique. (b) Schematic illustration of the IJP process and corresponding SEM images of SPCEs modified via IJP. (c) Photo of SPCE electrode subjected to profilometry measurement. (d) Profilometry measurements of SPCEs modified by DP and IJP, including the corresponding variance in the distribution of surface height. (e) AFM images of SPCEs modified by DP and IJP, including corresponding surface height profiles. (f) 3D topography images of SPCEs modified by DP and IJP. SEM characterization of a fully IJP electrode for dopamine detection printed on PET foil (g). Images of silver nanoparticle ink (used for contacts and reference electrode) (h) and gold nanoparticle ink (used for working and counter electrodes) (i) patterns show that silver and gold parts are made up of evenly distributed nanoparticles tens of nanometers in size. After IJP of the NGA‐ink onto already printed Au layer (j), the surface of the gold working electrode is also evenly covered with NGA flakes of approximately 300 nm in size (k, l). (a–f) Adapted under the terms of the CC‐BY 4.0 license [88]. Copyright 2025, Wiley‐VCH GmbH. (g–l) Adapted under the terms of the CC‐BY 4.0 license [24]. Copyright 2024, Elsevier.

In parallel with efforts to fabricate fully inkjet‐printed electrodes and devices, an alternative strategy is the functionalization of prefabricated screen‐printed electrodes (SPEs) using IJP. This hybrid approach integrates the scalability and simplicity of SP with the precision and versatility of inkjet deposition. Several studies have demonstrated the feasibility of this method for functionalizing carbon SPEs with nanomaterials such as graphene‐PEDOT:PSS [83] or graphene‐polyaniline [84] composites, polypyrrole [85] and Prussian blue [86] nanoparticles, graphene [76] and CNTs [87] modified with enzymes, and NGA [24]. Results have shown that this method enables uniform functionalization of the electrode surface and improves reproducibility compared to conventional deposition techniques, particularly DP [24, 88]. This integration of two printing techniques provides a practical and adaptable route for sensor development, particularly in settings where both large‐scale fabrication and fine‐tuned surface chemistry are desired. Microscopic analyses illustrating the uniformity of inkjet‐deposited functional layers on both screen‐printed and fully inkjet‐printed electrodes are presented in Figure 3.

Despite these advantages, several challenges remain for the IJP of nanomaterials. Common issues include coffee‐ring formation during drying, reduced film conductivity due to interparticle resistance, colloidal instability, and biocompatibility concerns related to ink components [89]. However, many of these limitations can be mitigated through careful control of ink formulation and process parameters. Strategies such as substrate heating, sintering, or photonic curing, layer‐by‐layer deposition, and the use of stabilizers or reactive binders have been shown to improve film uniformity, conductivity, and material integration [90]. A practical way to partially overcome the limited conductivity of some nanomaterials is to modify existing electrode surfaces with nanomaterial inks, avoiding the immediate need to develop fully standalone conductive formulations [88]. The shift toward water‐based, additive‐free inks also reflects a broader trend toward environmentally sustainable fabrication of electrochemical sensors [91].

Looking forward, advanced functional inks are expected to unlock new capabilities in printed sensor technologies. Inks with catalytic properties or smart inks containing bioactive, enzymatic, or stimuli‐responsive components hold potential for expanding the functionality of printed sensors beyond current limitations [8]. One particularly interesting approach to surface functionalization is reactive IJP, in which two or more inks are printed sequentially or simultaneously to initiate in situ chemical reactions. This approach has recently been used to produce complex modifications such as hydrogels [92, 93], conductive surfaces [94], micro stirrers [95, 96, 97], and metal–organic frameworks (MOFs) [98] with controlled stoichiometry, but has yet to be applied in electrode functionalization. If adapted for sensing, reactive IJP could enable the in situ fabrication of sensing interfaces that are otherwise unprintable in a single step, offering a new route for constructing multifunctional electrochemical sensing surfaces.

### 3.2 Comparative Analysis of Functionalized Electrode Performance Obtained via Inkjet, Drop‐Casting, or Screen Printing

**FIGURE 4:**
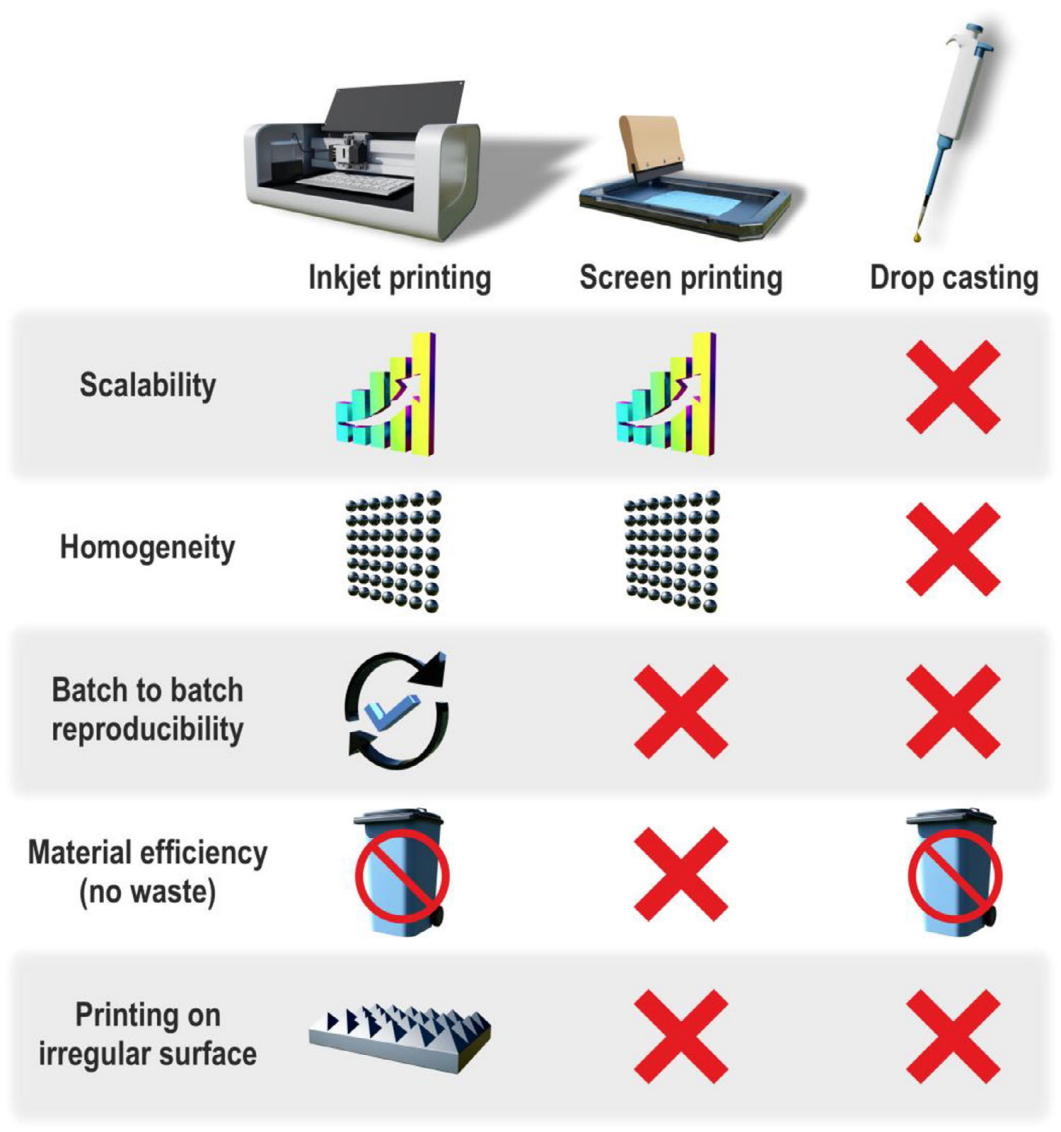
Comparison of IJP, SP, and DP for utilization in electrode functionalization.

The choice of deposition technique should be guided by critical performance criteria such as sensitivity, reproducibility, limit of detection (LOD), material efficiency, surface homogeneity, and suitability for miniaturization or batch production. IJP offers high material efficiency and precise control over film morphology, but remains constrained in scalability. SP supports large‐scale production but often yields thicker, less uniform layers. Its highly viscous inks require binders or agents that can mask active sites or interfere with bioreceptor function. DP is simple and accessible but suffers from poor reproducibility and inconsistent film formation. High‐surface‐area structures may boost sensitivity but have lower batch‐to‐batch consistency, while smoother layers improve reproducibility at the expense of signal strength. Understanding these structure‐function relationships is essential for optimizing sensor architecture and performance. Comparative advantages and limitations of IJP, SP, and DP are summarized in Figure 4, which highlights key differences in scalability, homogeneity, reproducibility, material efficiency, and adaptability to irregular surfaces.

The performance of (electro)chemical sensors is highly sensitive to the physical characteristics of the active layer—particularly its morphology, thickness, roughness, porosity, and uniformity. Each of these parameters, directly influenced by the deposition technique, plays a key role in determining sensitivity, reproducibility, and LOD.

Studies demonstrate that the active layer thickness significantly influences sensor performance, with optimal thickness varying according to application [99, 100, 101]. SP tends to produce thick layers that favor conductivity but can hinder sensitivity due to poor mass transport and increased diffusion barriers. Excessively thick layers may also elevate capacitive currents and baseline noise, thereby degrading detection limits [102, 103]. In contrast, IJP produces thinner, more uniform coatings that enhance mass transport and accessibility to the electroactive surface, leading to higher sensitivity and lower LOD [42, 61, 62, 104, 105]. Compared to DP, IJP significantly lowers both ohmic and diffusion resistances while increasing interfacial area and reaction sites, leading to improved electrocatalytic activity and sensor response [106]. Fine control over drop spacing further tailors morphology and film thickness, directly influencing sensor responsivity [107]. For instance, in humidity sensors, inkjet‐printed layers (∼2.5 µm) enabled faster moisture diffusion and signal response (∼0.8 s) compared to drop‐casted layers (∼17 µm), which were bulkier and with slower response [108]. Nonetheless, layers that are too thin may compromise conductivity and absolute signal strength due to insufficient electroactive material. Achieving an optimal intermediate thickness is crucial, balancing catalytic coverage with minimal diffusion limitations. IJP is particularly advantageous due to its precise control over layer thickness through drop spacing and multiple print passes, enabling tailored optimization for maximal sensor performance [109, 110, 111].

Morphological factors such as surface roughness, porosity, and electrochemical surface area (ECSA) further dictate device performance [112, 113]. A larger ECSA offers more redox‐active sites, enhancing signal strength, electron transfer rates, and sensitivity [114, 115]. For example, printing carbon ink on untreated rough paper increased ink penetration and ECSA, producing a current of 23 µA, which was about 2.7× higher than expected from the geometric area. However, this came with a trade‐off: rough surfaces yielded stronger signals but reduced reproducibility (relative standard deviation, RSD ∼3.6%), compared to smoother coated substrates, which gave lower signals (16 µA) but significantly better reproducibility (RSD ∼0.8%) [57].

Achieving reproducible and homogeneous electrode surfaces is essential for minimizing noise (and thus lowering the effective LOD) and reducing batch‐to‐batch variability. IJP excels in producing uniform, reproducible films, outperforming both DP and SP [116, 117, 118, 119, 120]. Inkjet‐printed sensors consistently show low single‐digit RSDs with superior electrocatalytic activity and electron transfer rates [110]. While increasing the electroactive surface area enhances peak current responses, it can also negatively affect reproducibility if not carefully controlled [114]. Nonetheless, the inherent consistency of IJP not only reduces within‐batch variability but also minimizes the need for individual sensor calibration within the same electrodes batch, thus saving time and resources [121]. In contrast, DP suffers from high variability (2–3× higher RSD) due to inconsistent film morphology and coffee‐ring effects [36, 122]. Although increased surface roughness or porosity can lower conductivity, the corresponding increase in surface area and active sites may compensate for this drawback [123].

## 4 Examples of Fully Printed Electrode Applications

IJP is highly effective for precisely and reproducibly modifying electrode surfaces, but it shows its greatest promise in fully inkjet‐printed sensors. In these sensors, all conductive and electroactive components, including the working, counter, and reference electrodes, are inkjet‐printed. This process enables high reproducibility, micrometer‐scale lateral resolution, and low manufacturing costs. A further refinement involves printing the dielectric components with insulating and dielectric inks. This step is crucial for isolating contacts, defining the sensing area, and creating patterns that can be used to perform sample transport, treatment, and pre‐analytical steps.

**FIGURE 5:**
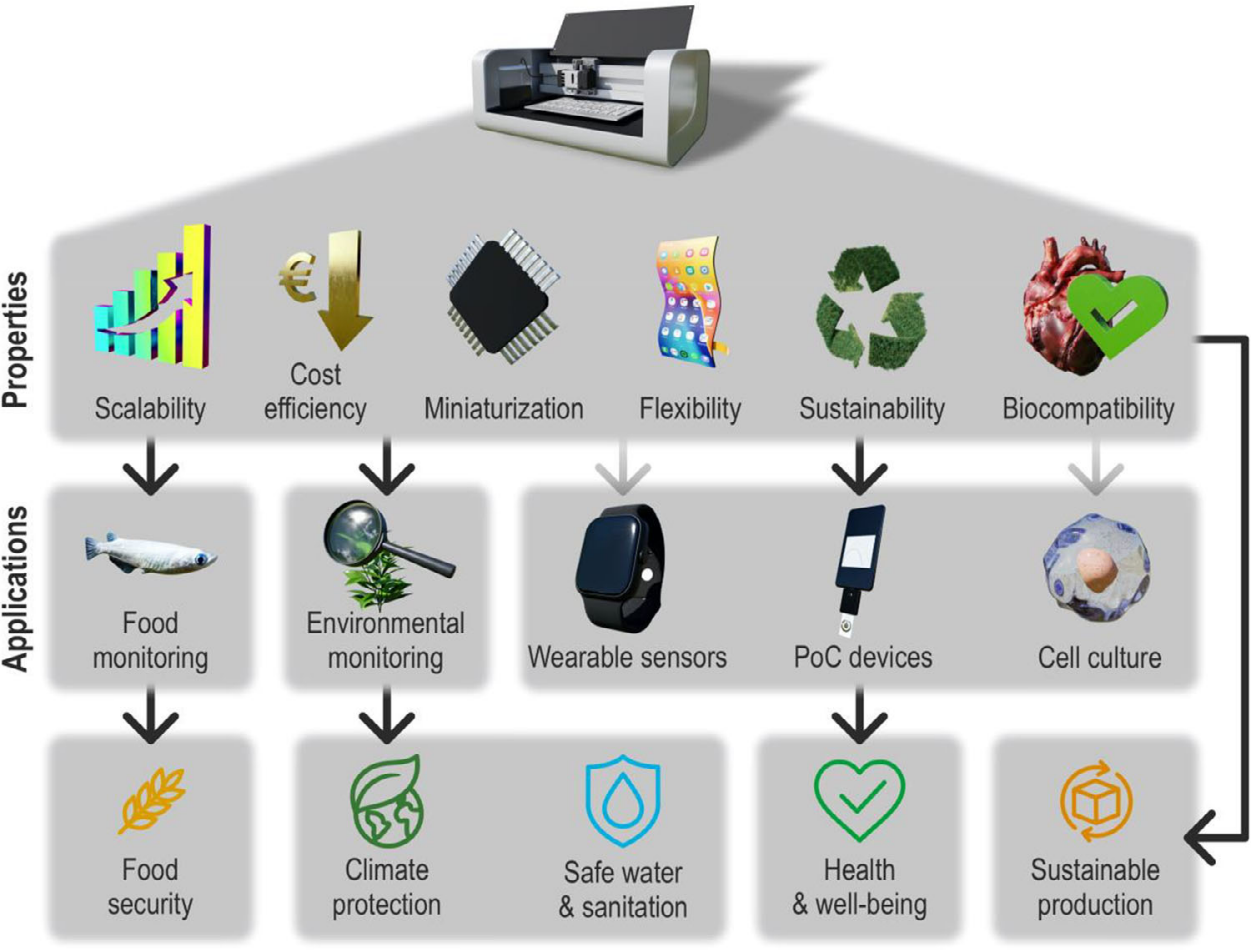
IJP electrodes find application in numerous fields, leveraging the intrinsic advantages of printed electrodes. IJP has the potential to address several of the UN's Sustainable Development Goals.

A few years ago, fully inkjet‐printed electrodes were primarily investigated for basic sensing applications such as temperature, humidity, gas, and pH detection. More recently, as technology has matured, researchers have begun to target more complex applications with significant societal impact. This shift leverages the intrinsic advantages of printed electrodes, including compatibility with a wide range of flexible substrates and the porosity and capillarity of these materials. In addition, the design and development of novel nanostructured inks are expanding the range of detectable analytes, while cost‐effective and scalable production make mass manufacturing feasible. When substrates and inks are carefully selected, IJP can also produce degradable and sustainable sensors with reduced environmental impact during both production and disposal. Collectively, these benefits position fully inkjet‐printed electrodes as a promising technology for disposable, wearable, and point‐of‐care (PoC) devices relevant to diverse applications in society (Figure 5).

Thanks to these characteristics, inkjet‐printed sensors contribute to several of the *UN's Sustainable Development Goals* (SDGs). Their use of eco‐friendly materials, such as paper, supports SDG 12 (Responsible Consumption and Production) by reducing waste and reliance on non‐biodegradable materials. The low cost and scalability of IJP advance SDG 3 (Good Health and Well‐being) by enabling accessible and affordable diagnostics for a broader population. Furthermore, the development of low‐cost, portable inkjet‐printed sensors for environmental monitoring and food quality assessment also supports SDG 6 (Clean Water and Sanitation), SDG 13 (Climate Action), and SDG 2 (Zero Hunger). This technology also meets the WHO REASSURED criteria by offering devices that are affordable, user‐friendly, and capable of rapid, sensitive, and specific analysis, making them suitable for low‐resource settings.

This perspective does not aim to provide a comprehensive overview of all published works on fully inkjet‐printed sensors. Instead, it highlights specific fields where we believe this technology can best demonstrate its potential. For an in‐depth review of the topic, readers are referred to previously published works [8, 23, 63, 104, 124].

### 4.1 Fully Inkjet‐Printed Electrodes in Wearable Technologies

IJP has the potential to be a transformative fabrication technique for wearable electronics, providing a robust route for creating highly functional, flexible, and stretchable devices. This capability has been extensively demonstrated by Gao and coworkers [11, 125, 126], who have developed advanced electrochemical sensors for sweat analysis, showcasing the versatility of IJP across various applications.

**FIGURE 6:**
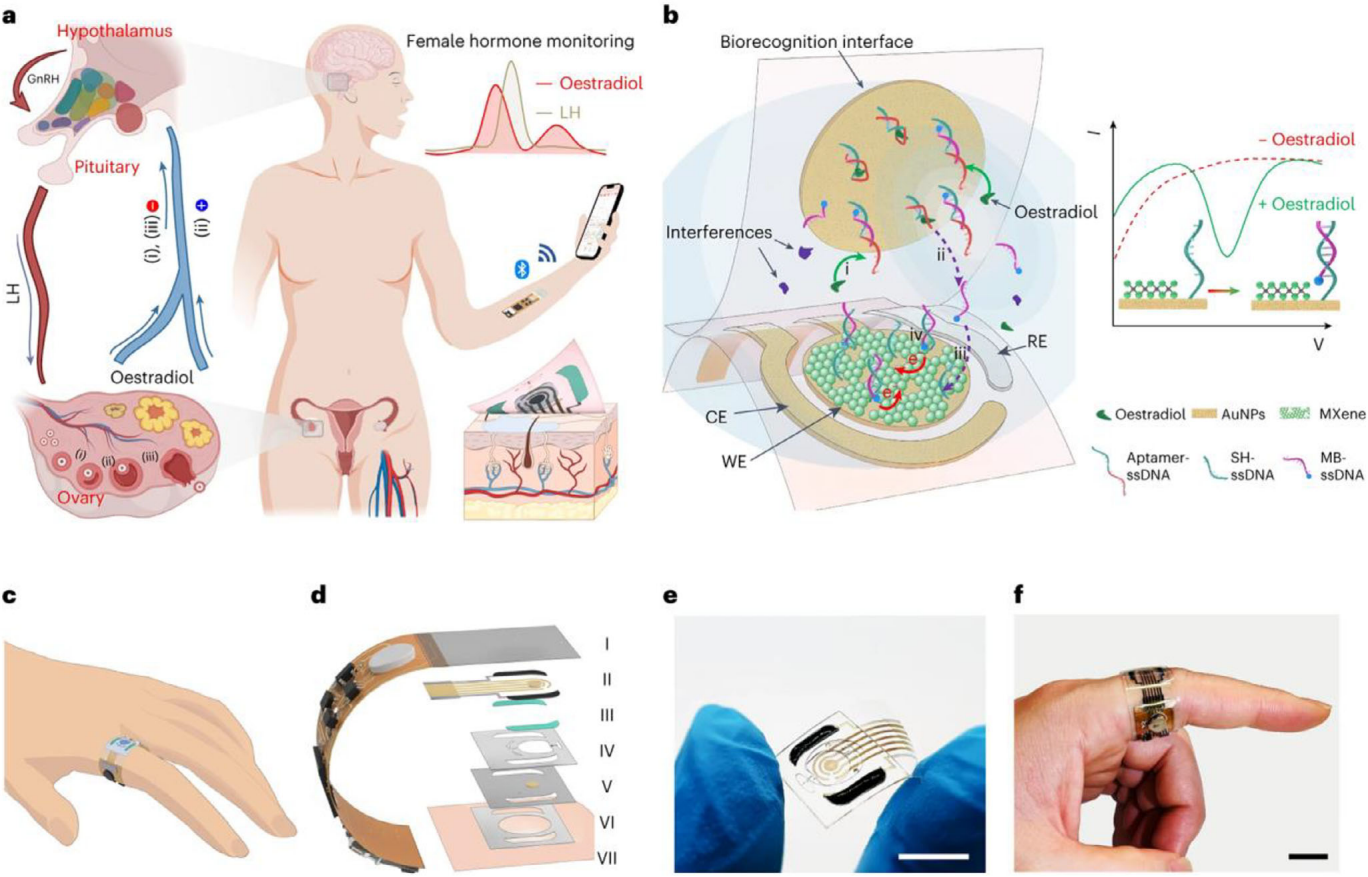
A wearable nanobiosensor based on a strand‐displacement aptamer switch for non‐invasive, reagentless female reproductive hormone analysis. (a) Female hormones play an important role in women's health; non‐invasive monitoring of oestradiol can be realized through sweat analysis using a skin‐interfaced wearable sensor: (i) follicular phase; (ii) ovulation; (iii) luteal phase. GnRH, gonadotropin‐releasing hormone; LH, luteinizing hormone; +, stimulatory effect; −, inhibitory effect. (b) The reagentless in situ quantification of oestradiol using AuNPs–MXene sensor coupled with a target‐induced strand‐displacement aptamer switch. i to iv represent recognition of the oestradiol molecule by the aptamer on the biorecognition interface (i), target recognition‐induced strand displacement to release the MB‐ssDNA (ii), recapture of the released MB‐ssDNA by the SH‐ssDNA on the working electrode (WE) (iii), and electrochemical quantification of the methylene blue from the recaptured MB‐ssDNA on the working electrode (iv). CE, counter electrode; RE, reference electrode. Schematic (c) and layered design (d) of the flexible and wireless microfluidic wearable patch for automatic sweat induction via iontophoresis, precise sampling via CBVs, and reagentless aptamer‐based oestradiol analysis. Sections I–VII represent plastic substrate interfacing with the FPCB (I), inkjet‐printed sensor array (II), carbachol‐loaded hydrogels (carbagels) (III), microfluidic channels (IV), biorecognition interface (V), sweat accumulation layer (VI), and skin (VII). Photographs of a disposable sensor patch (e) and a fully integrated wireless wearable patch worn on a finger (f) for female hormone monitoring. Scale bars, 1 cm. Reproduced with permission [126]. Copyright 2024, Springer Nature.

One example is the development of a sensor for gout management, where IJP enabled the fabrication of a multiplexed, mechanically flexible patch capable of detecting uric acid, xanthine, and alcohol in in vivo studies [125]. A key factor in its performance was the use of gold nanoparticle‐based inks, which produced WEs with a significantly larger ECSA, crucial for sensitive biomarker detection. In a separate study, Gao's group also developed a fully integrated wearable aptamer nanobiosensor for oestradiol monitoring, in which IJP played a fundamental role in the entire device design [126] (Figure 6). The system incorporated carbon‐based iontophoretic electrodes to autonomously stimulate and control sweat production, together with sensing electrodes printed from various nanomaterial inks such as gold, silver, and MXene.

Beyond specific applications, progress in developing smart and functional inks is further broadening the reach of wearable technologies. For example, printable core–shell nanoparticles, combining a molecularly imprinted polymer shell with a nickel hexacyanoferrate core, have enabled the scalable fabrication of flexible biosensors for monitoring a wide range of biomarkers, including amino acids, vitamins, and drugs [11]. This highlights how ink design directly contributes to the development of stable, versatile, and cost‐effective devices.

While sweat remains the most common target, IJP‐based wearable sensors are increasingly being adapted to other biological fluids, thereby expanding their diagnostic relevance. Skin‐conformable devices, for instance, have been developed to monitor nitric oxide (NO), hydrogen peroxide (H_2_O_2_), oxygen (O_2_), pH, and temperature in wound exudates [127]. Validated in both murine diabetic wound models and in twenty patients with chronic wounds, the system used machine learning to classify wound types and predict healing times. Complementing this direction, flexible multi‐sensor arrays have been integrated into face masks to monitor exhaled breath condensate [128]. This array can continuously monitor multiple biomarkers, including nitrite, alcohol, pH, and ammonium ions, while incorporating a tandem passive cooling strategy for condensation and a capillary‐inspired microfluidic module that enables gravity‐independent analysis even in supine positions. Clinical validation confirmed the system's usability for assessing metabolic conditions and airway inflammation in both healthy individuals and patients with chronic obstructive pulmonary disease (COPD), asthma, and post‐COVID‐19 infection.

Substrate selection also plays a critical role in exhaled breath analysis. For example, paper offers significant advantages as a low‐cost, disposable medium capable of capturing moisture and forming liquid electrolytic cells required for sensor operation [129]. Moreover, its cellulose fibers can be patterned with waxes, inks, or polymers to fine‐tune permeability, hydrophilicity, and reactivity, enabling absorption of fluids and their passive transport.

In parallel, IJP enables the fabrication of 3D architectures, such as microneedles for interstitial fluid analysis. Recently, Rosati et al. demonstrated a simple, scalable method for producing fully inkjet‐printed conductive microneedles from silver nanoparticle inks, with extensibility to other metallic systems [130]. The approach involves jetting ink droplets onto a heated substrate, curing them instantly to prevent spreading. This single‐step process overcomes many of the cost and infrastructure barriers associated with traditional microneedle fabrication, which typically requires expensive equipment, cleanroom facilities, or multi‐step processes.

Advances are not limited to sensing alone but extend to integrated fluid handling and signal processing. IJP offers a cost‐effective and scalable approach for producing 3D microfluidic devices that minimize material waste [131]. It also enables on‐demand hydrophilic coatings inside microchannels to regulate capillary flow, eliminating the need for bulky external pumps and valves [132]. Such developments point toward future platforms where sensing and fluidics are seamlessly integrated. At the same time, Gao and coworkers have extended IJP into neuromorphic architectures by proposing a chipless wearable sensor–processor capable of simultaneously monitoring and analyzing multimodal data [74]. The key elements of this device, including artificial synapses and nodes, were inkjet‐printed on flexible polymer substrates, enabling autonomous, efficient, and real‐time healthcare analysis. Such systems hold particular promise for early sepsis diagnosis and patient data classification.

Collectively, these examples demonstrate that IJP is not merely a manufacturing tool but a foundational technology for next‐generation wearable devices. It enables miniaturization and multiplexing of sensors on conformable platforms, supports integration with diverse nanomaterial inks, and provides scalability for mass production. Looking ahead, the field is evolving toward the monitoring of a broader spectrum of body fluids, including interstitial fluid, exhaled breath, and wound exudates, extending analysis beyond traditional biophysical measurements to metabolites, hormones, and proteins. While this evolution holds great promise, widespread adoption will require overcoming persistent challenges related to sensor accuracy, power supply, robust software, and, most critically, clinical translation and user acceptance [133].

### 4.2 Fully Inkjet‐Printed Electrodes as PoC for Biomedical Applications

Beyond wearable devices, IJP is a highly promising fabrication technique for disposable PoC devices, owing to its low production cost, scalability, and environmental sustainability. For instance, inkjet‐printed platforms have been successfully applied to detect biomarkers such as the breast cancer marker HER‐2 [134] and thyroid‐stimulating hormone (TSH) [135]. In these examples, IJP enabled the design of customized, user‐friendly, and cost‐effective sensors that simplified electrochemical ELISA assays by integrating multiple electrochemical cells on a single platform. This approach improved reproducibility and reduced operational time. Fabricated by printing conductive inks (gold, silver, CNTs) alongside dielectric inks onto flexible substrates, these devices cost less than $0.25 each, making them well‐suited for mass production.

Inkjet‐printed electrodes have also been extensively explored for detecting metabolites, as altered metabolite levels are often associated with pathological conditions. Glucose is the most widely studied compound, frequently serving as a benchmark for validating new sensor technologies due to its critical role in diabetes monitoring and a well‐established detection mechanism [77, 114, 136]. This mechanism can rely on direct electrochemical oxidation under alkaline conditions or on enzymatic reactions involving glucose oxidase. Importantly, IJP allows enzymes such as glucose oxidase to be incorporated directly into inks, either in their native form [137], chemically or physically stabilized to enhance activity [91], or bioconjugated with other materials to improve efficiency of the electrochemical reaction [76]. In addition to glucose, enzyme‐based inks have been developed for lactate, alcohol, cholesterol, glutamate, choline, and bilirubin, further expanding the scope of PoC diagnostics.

Moving beyond metabolites, neurotransmitters represent another clinically important class of analytes for diagnosing and monitoring neurodegenerative, neuropsychiatric, and stress‐related diseases. Fully inkjet‐printed PoC devices for neurotransmitter detection have been reported using inks based on graphene derivatives [24], boron‐doped diamond [138], and CNTs [99]. These nanomaterials provide a large surface area, high conductivity, and strong electrocatalytic properties, which are particularly advantageous for catecholamine detection, such as dopamine. While dopamine remains the most explored target, inkjet‐printed electrodes also show promise for epinephrine, norepinephrine, serotonin, glutamate, and acetylcholine. Furthermore, by using biocompatible and biodegradable substrates such as silk [139], electrodes can be integrated into implantable sensors or scaffolds for neuronal cultures, offering opportunities for both in vivo monitoring and in vitro disease modeling or drug screening.

**FIGURE 7:**
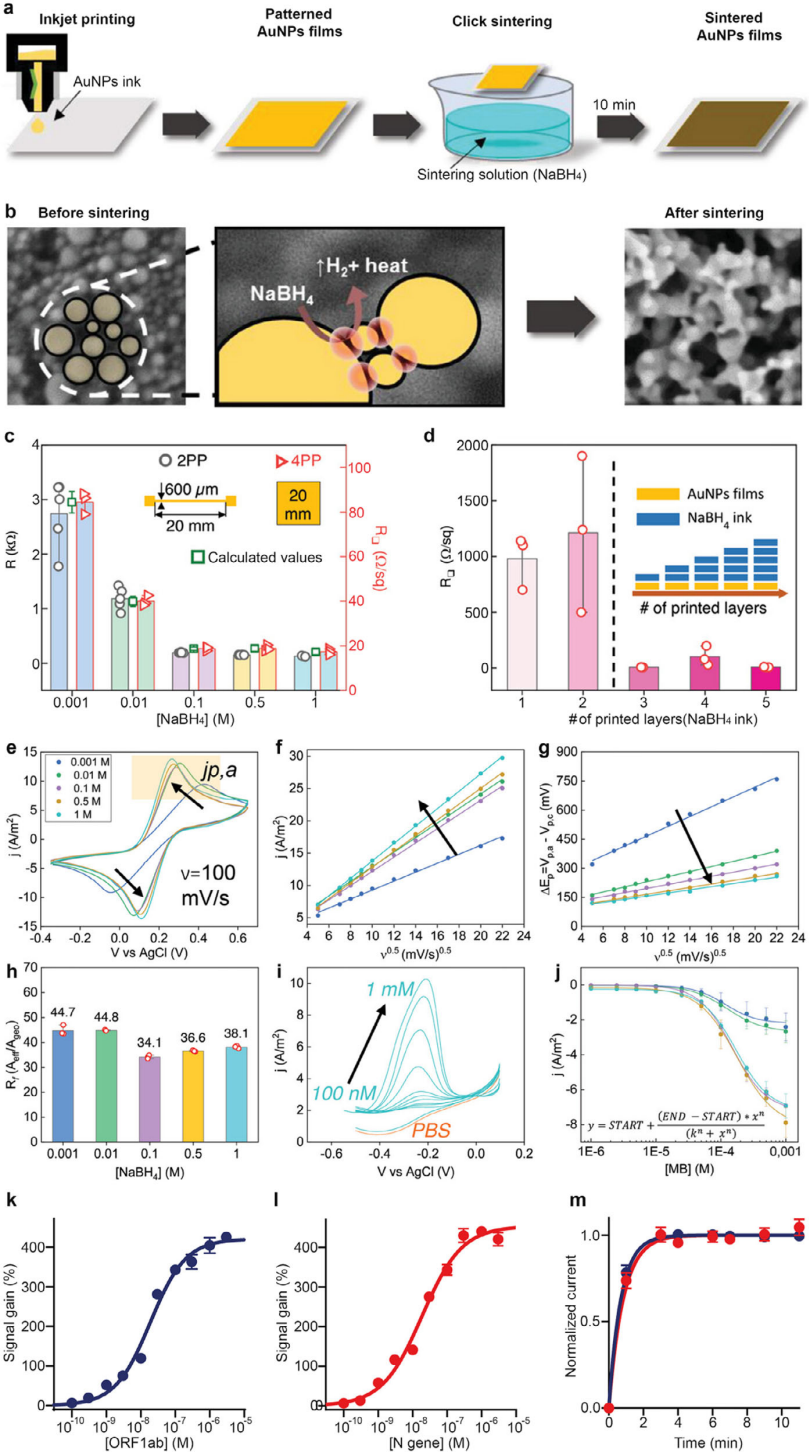
Nanostructure tuning of gold nanoparticle films via click sintering enables the integration of inkjet‐printed electrodes with a battery‐free potentiostat for multiplexed DNA‐based detection of SARS‐CoV‐2 genes. (a) Scheme of generic printing and sintering protocol for AuNPs inkjet‐printed films. (b) Illustrative representation of the sintering mechanism involving the reaction with the sintering agent (NaBH_4_) and the local nanoenvironment at the interphase. (c) Sheet resistance values for the AuNPs‐ink printed squares (in red) and resistance values of the printed lines (in black), after the 10 min treatment with different concentrations of NaBH_4_; the squares represent the calculated resistance using the sheet resistance and the aspect ratio of the trace. (d) Sheet resistance values for the 20 mm squares, after real‐time inkjet sintering on Kapton at increasing numbers of layers, showing a threshold in the relative amount of sintering solution (NaBH_4_ 0.5 m ink). The columns represent the average values, and the bars represent the respective standard deviations (n = 3). Electrochemical characterization of the treated electrodes. (e) Cyclic voltammetry measurements were performed on 4 mm square AuNPs printed working electrodes with an external Ag/AgCl reference and Pt counter electrodes in 5 mm ferro/ferricyanide in 10 mM phosphate saline buffer (PBS, pH 7.4, 23°C). (f) Anodic peak currents recorded vs. the scan rate for the electrodes treated with different concentrations of NaBH_4_. (g) Peaks separation as a function of the scan rate for the treated electrodes. (h) Roughness factor of the films at the different concentrations of the chemical agent. (i) Square wave voltammetry (SWV) response of the films treated with 1 m NaBH_4_ for concentrations of MB in PBS ranging from 100 to 1 mm. (j) Calibration curve fitted with a Hill equation (inset) for the films treated with the different concentrations of the sintering agent (n = 3). SWV parameters: 25 Hz frequency, 25 mV amplitude, potential range from 0.1 to −0.4 V. Quantitative simultaneous detection of synthetic fragments of (k) ORF1ab gene and (l) N gene. (m) Kinetics of hybridization when the sensor is tested with a saturating concentration (i.e., 3 µm) of target (i.e., ORF1ab in blue, N gene in red). (a–j) Adapted with permission [141]. Copyright 2024, Wiley‐VCH GmbH. (k–m) Adapted with permission [61]. Copyright 2024, Elsevier.

Nucleic acid detection has also benefited from the use of IJP. Early work by Ihalainen et al. demonstrated the use of paper‐based, inkjet‐printed gold nanoparticle electrodes for DNA hybridization in the picomolar range, utilizing electrochemical impedance spectroscopy [140]. While promising, these systems face challenges due to the impedimetric response being strongly influenced by the limited conductivity of printed traces, nanostructured electrode roughness, fragile contacts, and electrolyte penetration into the paper. More recent advances have addressed these limitations. For instance, a platform that incorporated an innovative click sintering method to increase surface area was coupled with a battery‐free NFC potentiostat for smartphone‐based real‐time analysis (Figure 7a–j) [141], and it was subsequently utilized by Rossetti et al. to design a DNA‐based point‐of‐care platform with inkjet‐printed nanostructured gold electrodes capable of simultaneously detecting two SARS‐CoV‐2 genes (ORF1ab and N) in accordance with WHO diagnostic recommendations (Figure 7k–m) [61]. The same nanostructured gold electrodes were further employed by Carota et al. in a CRISPR/Cas12a system for detecting DNA sequences from clinically isolated samples of pathogenic bacteria, including *Escherichia coli* and *Staphylococcus aureus* [42].

The versatility of IJP extends beyond electrodes into the fabrication of transistor‐based biosensors. Between 2022 and 2023, the first fully inkjet‐printed, flexible electrolyte‐gated organic FETs (EGOFETs) were reported [142, 143]. For instance, Chennit et al. fabricated an EGOFET on polyimide using gold nanoparticles and p‐type semiconducting inks, achieving precise material deposition without protective layers or lithography [142]. Similarly, Demuru et al. created organic electrochemical transistors (OECTs) entirely by IJP using graphene, PEDOT:PSS, and silver inks, yielding devices with excellent electrical performance and high sensitivity [143]. Further refinements, such as the “surface‐tension‐guided drop‐and‐spread” technique [144], have improved the alignment and density of CNTs within transistor channels, enabling highly sensitive serotonin detection at thresholds as low as 42 pM.

Despite these advances, the most significant challenge for PoC devices remains reliable operation in complex biological fluids. Clinical samples such as blood, urine, and saliva contain diverse components, including cells, proteins, and lipids, which can interfere with sensor specificity and performance. One strategy to mitigate this is to exploit the intrinsic properties of the substrate. Paper, for instance, provides porosity that acts as a passive filter, physically removing particulates and cells while simultaneously preconcentrating analytes through solvent evaporation [145]. Its capillary action enables autonomous fluid transport without the need for external pumps, and its foldability allows for the creation of origami‐, plug‐and‐play, or pop‐up‐style devices with integrated pretreatment zones. Harnessing such substrate properties is a promising path for bridging laboratory‐based research with clinical translation of printed PoC technologies.

### 4.3 Fully Inkjet‐Printed Electrodes for Food and Environmental Monitoring

IJP sensors offer a powerful solution for analytical applications in food and environmental monitoring due to their core advantages: low cost, large‐scale production, and portability. These characteristics enable a shift from traditional laboratory‐based analysis to a decentralized model, allowing for in situ and frequent testing even in remote or resource‐limited locations. However, only a limited number of printed sensors for these applications have been reported so far, most of which are colorimetric [146, 147]. While colorimetric sensors are easy to read with the naked eye, they often fail to meet the sensitivity and low LOD required for regulated compounds. This gap highlights the need for further development of electrochemical IJP electrodes, which can combine portability with high analytical performance.

**FIGURE 8:**
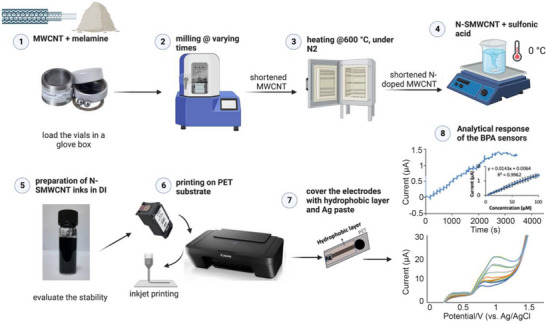
Schematic representation of the sensor fabrication and bisphenol A detection. Reproduced with permission [148]. Copyright 2023, Elsevier.

Food quality and safety have been an early testing ground for such devices. For example, Sarıkaya et al. developed a flexible, low‐cost electrochemical sensor using IJP of multi‐walled CNTs to detect bisphenol A (BPA) in milk (Figure 8) [148]. BPA is a widespread endocrine disruptor regulated in many countries, and food packaging is often the primary source of contamination. Similarly, Cinti et al. designed paper‐based IJP electrodes for ascorbic acid detection in dietary supplements, achieving excellent recovery. The performance of these electrodes was further enhanced by modifying them with carbon black [149]. Together, these studies illustrate how IJP devices can address relevant food‐related analytes with both sensitivity and affordability.

Environmental monitoring provides another domain where IJP devices are highly attractive, particularly for decentralized, on‐site detection of pollutants. Notable examples include a paper‐based PEDOT:PSS/zinc oxide electrode for detecting the toxic pollutant hydrazine in water samples [150] and a silver electrode printed on chromatographic paper for the detection of pesticide paraquat [151]. These demonstrations highlight how fully printed electrochemical devices can function as efficient, low‐cost tools for real‐time environmental surveillance.

Despite these advances, the transition from controlled laboratory settings to unpredictable field applications presents significant challenges. Reproducibility, mechanical integrity, and long‐term stability of the printed materials remain significant hurdles, as they are challenging to guarantee outside of the laboratory. Nonetheless, IJP offers distinct strategies to address these limitations. For instance, a Nafion encapsulation layer can be printed directly onto the electrode to enhance stability and protect the active materials [150]. Additionally, the digital precision of IJP ensures high patterning accuracy, contributing to reproducible device performance. Finally, the ability to fabricate disposable, single‐use sensors helps eliminate the “memory effect” caused by surface contamination, thereby ensuring accurate readings with each measurement.

## 5 New Trends and Opportunities

Recent developments in smart wearables, health monitoring devices, and soft robotics have significantly accelerated interest in printed physicochemical sensors on flexible and stretchable substrates [152, 153, 154]. While printing electrodes on flexible substrates like polyethylene terephthalate (PET) [155, 156], polyethylene (PE) [157] or polyimide (PI) [74] is still common due to their stability and established processing, there is a clear emerging trend toward stretchable elastomers such as polydimethylsiloxane (PDMS), polyurethane (PU)‐based elastomers, styrene‐ethylene‐butylene‐styrene (SEBS) and other thermoplastic elastomers [158, 159]. These stretchable materials offer superior conformability, durability under dynamic deformation, and comfort for direct skin integration, which are essential features for applications in sports performance tracking, medical diagnostics, and disposable healthcare sensors. A further step toward sustainability involves the adoption of biodegradable substrates such as paper and polylactic acid (PLA). Yet, these eco‐friendly materials often lack the mechanical and surface properties required for high‐quality IJP, underlining the need for additional research to optimize them for advanced printed electronics. Bridging this demand, techniques such as inkjet [160, 161], 3D/material [162, 163, 164, 165, 166], and screen [167, 168] printing on elastomers are increasingly employed to meet demands for lightweight sensors with high sensitivity, despite ongoing challenges related to ink compatibility, adhesion, and durability under repeated deformation. This shift toward elastic substrates, therefore, represents a major opportunity for innovation, especially in niche, high‐value markets such as personalized health monitoring, sport diagnostics, and soft robotic interfaces.

In parallel, the development of sustainable printed electronics is gaining traction, aiming to replace traditional metallic inks with eco‐friendly alternatives. This transition involves using organic and biodegradable conductive inks, such as those based on carbon derivatives or conductive polymers, instead of precious metals like silver and gold. A particularly significant challenge in this context is finding a replacement for the silver/silver chloride (Ag/AgCl) redox couple in reference electrodes. Although Ag/AgCl electrodes provide stable and reproducible potential, their reliance on silver and the need for a chloride‐saturated electrolyte make them unsuitable for disposable, sustainable applications.

Ink formulation is another crucial frontier. Developing water‐based conductive inks for IJP would markedly reduce environmental impact compared with conventional solvent‐based systems that release harmful volatile organic compounds. However, such inks often exhibit rheological properties unsuitable for reliable jetting, requiring the addition of carefully chosen additives, surfactants, and co‐solvents. In DOD printing, droplet formation depends strongly on viscosity, surface tension, density, and solvent volatility, which together determine whether a stable filament can detach from the nozzle [169]. For most printheads, the viscosity window of approximately 1–20 mPa·s and a surface tension around 20–40 mN·m^−1^ support consistent droplet generation [170, 171]. These parameters are often described using the inverse Ohnesorge number (Z), which provides a practical guide for assessing printability. Although a commonly cited printable range is 1< Z < 14 [172], nanomaterial inks, such as water‐based graphene dispersions [24, 88], have demonstrated good jetting behavior even outside these limits due to shear‐thickening or stabilizing effects introduced by the dispersed phase. As a result, formulation strategies that balance colloidal stability with the fluid properties required for drop formation remain central to the development of robust functional inks for sensing applications.

Taken together, these examples demonstrate that IJP sensors are rapidly evolving, enabling the development of next‐generation technologies. The versatility of this technique opens up pathways to futuristic applications, such as those already reported, including miniaturized, inkjet‐printed electrodes embedded within ingestible capsules for continuous biochemical profiling of the gastrointestinal tract [173].

## 6 Summary and Outlook

IJP has reached a turning point, evolving from a fabrication method into a cornerstone of the new era of additive, digital, and sustainable sensor technology. Its ability to precisely deposit nanomaterials, enzymes, or conductive polymers onto a variety of substrates allows it to bridge the gap between laboratory research and real‐world application. IJP offers unmatched control over surface functionalization, enabling reproducible electrode architectures that were once limited to lithographic techniques but are now achievable in open, low‐cost environments.

Yet, realizing its full potential requires addressing persistent challenges. The stability of inks, their compatibility with diverse printheads, and their adhesion to unconventional substrates remain key technical bottlenecks. The discovery of new printable materials continues to expand the palette of functional inks available for inkjet printing, broadening the possibilities for scalable device manufacturing. At the same time, it remains equally important to refine and improve the production routes of established materials, like for instance carbon and graphene‐based nanomaterials. These materials can now be synthesized at large scale using robust and well‐established protocols [174, 175, 176, 177, 178, 179], making their integration into printing workflows increasingly practical. Once produced, they can be formulated into stable inks, offering a cost‐effective alternative to precious‐metal formulations. In many cases, the use of graphene‐based inks can reduce material‐related device costs by more than an order of magnitude compared to metallic nanoparticle inks, significantly improving the economic feasibility of large‐scale inkjet‐printed sensor production. The development of predictive models, AI‐assisted process control [180], and new ink chemistries, particularly water‐based, biocompatible, and biodegradable formulations, will be essential for pushing IJP into fully autonomous, sustainable manufacturing.

The future landscape of IJP‐enabled sensors is both diverse and dynamic. Wearable and implantable platforms extend sensing capabilities beyond physical parameters to include complex biochemical markers such as metabolites, hormones, and proteins across various body fluids. Similarly, the use of flexible and porous substrates like paper or elastomers introduces novel functionalities: passive fluid handling, analyte preconcentration, and foldable architectures for plug‐and‐play or origami‐style devices. These advances could transform point‐of‐care systems, particularly when combined with wireless data transmission, self‐powered operation, and diagnostics driven by machine learning.

However, translation to clinical and field applications still faces substantial challenges: maintaining specificity and stability in complex biological fluids, ensuring reproducibility under real‐world conditions, and building user trust through reliable and validated performance. The reproducibility of the sensing platform is arguably the most critical requirement for any analytical device. As a reference, the current state‐of‐the‐art benchmark can be drawn from commercial glucometers. According to the FDA, an acceptable variation in performance between devices is approximately 5%, and for glucose determination in whole blood, an accuracy of ±20% across the physiological range of 1–30 mm is required [181]. Achieving comparable levels of reproducibility in inkjet‐printed sensors will require rigorous control over the entire fabrication workflow, from ink formulation and its physical stability to consistent jetting behavior during printing, and finally to post‐processing steps such as curing or sintering. Only by ensuring reproducibility at each stage of production can inkjet‐printed platforms meet the analytical reliability demanded for real‐world diagnostic applications. IJP offers promising routes to address these barriers, for example, by enabling reproducible, digitally defined encapsulation layers that improve stability, or through disposable architectures that eliminate contamination and memory effects.

Looking ahead, IJP is poised to become a foundational technology for next‐generation sensing, uniting precision manufacturing, sustainability, and intelligent design. By merging advances in materials chemistry, digital fabrication, and data analytics, IJP can transform how we imagine, fabricate, and deploy sensors, paving the way toward truly integrated, autonomous, and environmentally responsible diagnostics and monitoring systems.

## Author Contributions

D.P. contributed to conceptualization, writing of the original draft, review and editing, and visualization. I.D., M.‐A.N., M.U., A.S., A.M., and M.P. contributed to the writing of the original draft and to the review of the manuscript. M.O. contributed to conceptualization and methodology, as well as to review and editing of the manuscript, funding acquisition, and supervision.

## Declaration of Generative AI in Scientific Writing

While preparing this work, the authors used ChatGPT to improve the readability and language of the manuscript. After using this tool, the authors carefully reviewed and edited the content as needed, taking full responsibility for the accuracy and content of the publication.

## Conflicts of Interest

M.O. discloses his share in InSiliBio (France), focused on biosimulations, and ATOMIVER (Czechia), focused on supercapacitor electrode materials, companies.
